# Real-world analysis of acamprosate use in patients with cirrhosis and alcohol-associated hepatitis

**DOI:** 10.1136/bmjgast-2024-001654

**Published:** 2024-12-22

**Authors:** Christopher Oldroyd, Jonathan Wood, Michael Allison

**Affiliations:** 1 University of Cambridge, Cambridge, Cambridgeshire, UK; 2 Cambridge University Hospitals NHS Foundation Trust, Cambridge, UK; 3 Cambridgeshire and Peterborough NHS Foundation Trust, Fulbourn, Cambridgeshire, UK; 4 NIHR Cambridge Biomedical Research Centre, Cambridge, Cambridgeshire, UK

**Keywords:** ALCOHOL, CIRRHOSIS, HEPATITIS, ALCOHOLIC LIVER DISEASE

## Abstract

**Objective:**

Preventing return to alcohol is of critical importance for patients with alcohol-related cirrhosis and/or alcohol-associated hepatitis. Acamprosate is a widely used treatment for alcohol use disorder (AUD). We assessed the impact of acamprosate prescription in patients with advanced liver disease on abstinence rates and clinical outcomes.

**Methods:**

This was a retrospective case–control study. We reviewed data on all patients admitted to a large tertiary centre in the UK with alcohol-related cirrhosis and/or alcohol-associated hepatitis. We used propensity risk score matching to match patients prescribed acamprosate to controls. The primary outcome was repeat hospitalisation.

**Results:**

There were 451 patients who met the inclusion criteria of whom 55 patients were started on acamprosate during their admission. Before matching there were significant differences between the cohorts. Patients who received acamprosate were younger (median age 51 vs 57, p<0.005), more likely to have a purely alcohol-related admission (53% vs 24%, p<0.001), and more likely to suffer from a comorbid psychiatric diagnosis (42% vs 20%, p<0.001). On average patients who were started on acamprosate consumed more alcohol (median 155 units/week vs 80 units/week, p<0.001), were less likely to have a partner (35% vs 54%, p 0.006) and more likely to be unemployed (67% vs 44%, p<0.001). After matching for factors with significant differences between groups, we generated a cohort of 53 patients prescribed acamprosate and 53 matched controls. At 1 year there was a significantly higher rate of readmission (85% vs 57%, p<0.001) in the acamprosate group. There were no statistically significant differences in abstinence rates or mortality at 1 year.

**Conclusion:**

Acamprosate prescription was associated with higher rates of readmission in patients with cirrhosis and/or alcohol-associated hepatitis. This may reflect a greater severity of AUD in those patients or might indicate the limited ability of acamprosate to alter the disease course in this population.

**WHAT IS ALREADY KNOWN ON THIS TOPIC**

- Acamprosate is an effective treatment for the prevention of return to alcohol. This intervention has received very little study in patients with alcohol-related cirrhosis and/or alcohol-associated hepatitis.

**WHAT THIS STUDY ADDS**

- Acamprosate prescription was associated with higher rates of hospital readmission and no benefit in mortality or abstinence at 1 year for patients with cirrhosis and/or alcohol-associated hepatitis.

**HOW THIS STUDY MIGHT AFFECT RESEARCH, PRACTICE, OR POLICY**

- Although safe, this study questions the effectiveness of acamprosate in this population. This question might be answered with a well-designed randomised trial.

## Introduction

 Alcohol use is the leading cause of cirrhosis globally, and cases of alcohol-related liver disease (ArLD), including alcohol-associated hepatitis, continue to rise in most parts of the world.^1^ Abstinence from alcohol is the most important intervention for patients with advanced ArLD. Indeed, abstinence from alcohol improves clinical outcomes at all stages of liver disease.^2 3^ The impact of abstinence on mortality is particularly stark in cases of alcohol-associated hepatitis.^4 5^ Despite this, relapse rates and alcohol use among this population remain high.^3 6^ The evidence base for any intervention for alcohol use disorder (AUD) in patients with alcohol-related cirrhosis remains poor,^7^ and uptake of treatments is low.^8^ Hospital admission creates an enforced period of abstinence and should represent an opportunity for patients and healthcare professionals to work together to attempt to prevent future relapse to alcohol.

Acamprosate is a widely used treatment for AUD in patients without liver disease. It is recommended as a first-line measure to prevent relapse in patients who have achieved abstinence from alcohol and should be prescribed in conjunction with psychological interventions.^9 10^ The pharmacology of acamprosate is not completely understood but it is thought to act as a glutaminergic antagonist and a γ-aminobutyric acid agonist. It is suggested to reduce craving by reducing arousal, insomnia and anxiety.^11^

In outpatient settings, the number needed to treat to prevent a return to drinking for acamprosate in one patient is 12.^12^ There have been no randomised studies of acamprosate in patients with cirrhosis or alcohol-associated hepatitis. Nevertheless acamprosate is broadly considered to be safe in this population since it is renally excreted and has no known hepatotoxicity.^13 14^ It is therefore an attractive candidate for use in patients with cirrhosis or alcohol-associated hepatitis for whom relapse prevention is critical.

In a recent retrospective analysis of patients with cirrhosis, acamprosate was not found to be associated with any significant adverse events and was associated with fewer hospital admissions than baclofen.^15^ However, other cohort studies have demonstrated that patients with AUD who had been treated with acamprosate were more likely to be diagnosed with ArLD and to develop hepatic decompensation.^16^ Moreover, even in patients without liver disease, acamprosate prescription has been associated with an increased risk of hospitalisation.^17^

We wanted to determine the real-world impact of acamprosate prescription for patients with alcohol-related cirrhosis or alcohol-associated hepatitis following a hospital admission. We were interested not only in the impact of acamprosate on relapse to alcohol, but also in any impact on clinical outcomes.

## Methods

This was a retrospective case–control study. We reviewed data on all patients admitted to a large tertiary centre in the UK with alcohol-related cirrhosis and/or alcohol-associated hepatitis. We included patients who might be candidates to be prescribed acamprosate as relapse prevention during their admission or at the point of discharge. Therefore we included all patients with a confirmed diagnosis of alcohol-related cirrhosis or alcohol-associated hepatitis, who had an emergency admission to hospital lasting>24 hours and were drinking alcohol at the time of presentation. Diagnosis of cirrhosis was based on clinical documentation substantiated by any of the following: imaging, biopsy, blood results consistent with cirrhosis, evidence of portal hypertension or overt hepatic decompensation. Reasons for admission were categorised as ‘Liver’ (decompensated cirrhosis and alcohol-associated hepatitis), ‘Alcohol’ (alcohol-related admission without evidence of hepatic decompensation, for example, alcohol withdrawal syndrome, alcohol-related pancreatitis), and ‘Other’ (no link to alcohol or liver disease). Where patients had multiple admissions during the study period, the admission on which they were prescribed acamprosate was used. For those not prescribed acamprosate, the first admission in the study period was used. We excluded patients who did not survive their index admission. Admissions were reviewed between 1 October 2014 and 1 March 2023.

Data was extracted from electronic health records. Each case was individually reviewed by a hepatologist. In addition to basic demographics, we collected data on social status, marital status, living status and comorbidities, since it was predicted that these variables might influence outcomes and/or acamprosate prescription. We also collected baseline liver disease severity scores, specifically United Kingdom Model for End Stage Liver Disease (UKELD) and Model for End Stage Liver Disease (MELD).

All outcomes were assessed at 1 year following admission. The primary outcome was all-cause hospital readmission. Readmission rate was defined as the number of patients readmitted at least once during follow-up compared with the total patients with data available. Secondary outcomes were abstinence from alcohol and mortality. We also collected data on liver disease severity scores, clinic attendance, alcohol service engagement and alcohol use at 1 year. Engagement with alcohol services was defined as any documented contact with community alcohol services or alcohol support groups within 1 year of discharge. This included the use of fellowship organisations such as Alcoholics Anonymous. Clinic non-attendance was defined as failure to attend>2 appointments without cancellation or explanation. Outcome measures were chosen following consultation with a patient advisory panel with lived experience of AUD and alcohol-related cirrhosis. Following a preliminary analysis of our data set we estimated a recruitment of 81 cases to have an 80% chance of detecting a difference in 1 year readmissions between groups of 20% with a confidence of 95%.

All data analysis was completed on RStudio V.2023.06.1+524. Propensity score matching was used to account for variables which were likely to influence the prescription of acamprosate and to match each treated case to control. Propensity score analysis is a causal inference technique for treatment effect estimation in observational studies which reduces bias by accounting for the probability of treatment selection. The matching method used was one-to-one nearest neighbour. This involves minimising the absolute difference between the estimated propensity scores of each matched pair. A randomly selected patient who received acamprosate was matched with a control who had the propensity score closest in value. Propensity scores were estimated using logistic regression. Matching was achieved using the ‘Matchit’ function in R. Variables for the logistic regression model were initially selected as those which showed statistically significant differences between treated and untreated patients in the unmatched population and which might influence the decision to prescribe acamprosate.

After matching there were no statistically significant differences in baseline data between cases and controls. Therefore no multivariate analysis of outcomes was undertaken. Continuous variables were compared using the Wilcoxon rank sum test. Categorical variables were compared using χ² test or Fisher’s exact test (where any cell count<5). Throughout the analysis, missing data were handled using pairwise deletion. This approach maximises the use of available data for each analysis but may result in different sample sizes for different analyses. Those sample sizes are evident from the tables.

The study was sponsored by Cambridge University Hospitals National Health Service Foundation Trust. We reported our findings according to the Strengthening the Reporting of Observational Studies in Epidemiology guidelines (online supplemental material 1).

## Results

### Unmatched cohort

There were 451 patients who met the inclusion criteria of whom 55 patients were started on acamprosate during their admission. In these patients the median age was 56 and 63% were male. The median MELD score was 18. No other pharmacotherapies were prescribed for AUD across the cohort.

Before matching there were significant differences between the cohorts (table 1). Patients who received acamprosate were younger (median age 51 vs 57, p<0.005). These patients were more likely to have a purely alcohol-related admission (53% vs 24%, p<0.001), rather than an admission due to liver disease, and more likely to suffer from a comorbid psychiatric diagnosis (42% vs 20%, p<0.001). On average patients who were started on acamprosate were drinking more alcohol on admission compared with those who did not (median 155 units/week vs 80 units/week, p<0.001). Finally, patients prescribed acamprosate were less likely to have a partner (35% vs 54%, p0.006) and more likely to be unemployed (67% vs 44%, p<0.001).

**Table 1:** Unmatched data

	Overall, n=451*	Controls, n=396*	Acamprosate, n=55*	P value†
Age	56 (49–65)	57 (50–66)	51 (43–57)	<0.001
Female	165 (37)	146 (37)	19 (35)	0.7
Reason for admission				<0.001
Alcohol	125 (28)	96 (24)	29 (53)	
Liver	253 (56)	234 (59)	19 (35)	
Other	73 (16)	66 (17)	7 (13)	
Alcohol-associated hepatitis	113 (25)	101 (25)	12 (22)	0.6
Admission alcohol intake (units/week)	90 (50–150)	80 (40–143)	155 (100–280)	<0.001
Living status				0.9
Alone	159 (36)	139 (36)	20 (37)	
Not alone	281 (64)	247 (64)	34 (63)	
Marital status				0.006
Partner	210 (52)	191 (54)	19 (35)	
No partner	196 (48)	160 (46)	36 (65)	
Employment status				<0.001
Employed	103 (25)	89 (24)	14 (25)	
Retired	118 (28)	114 (31)	4 (7.3)	
Unemployed	199 (47)	162 (44)	37 (67)	
Psychiatric comorbidity	101 (22)	78 (20)	23 (42)	<0.001
Length of stay (days)	5 (3–11)	6 (3–12)	4 (2–9)	0.024
MELD	18 (14–23)	18 (14–23)	17 (13–20)	0.10
Review by liaison psychiatry	314 (70)	264 (67)	50 (93)	<0.001
Outcomes at 1 year				
No of admissions	1 (0–2)	1 (0–2)	3 (1–4.25)	<0.001
Readmitted	203 (57)	162 (52)	41 (85)	<0.001
MELD	13 (11–17)	13 (11–17)	13 (11–17.8)	>0.9
Abstinence				0.007
Abstinent	144 (47)	133 (50)	11 (28)	
Not abstinent	161 (53)	132 (50)	29 (73)	
Mortality				0.4
Dead	82 (19)	75 (20)	7 (14)	
Alive	345 (81)	303 (80)	42 (86)	
Community alcohol service use				<0.001
No	123	117	6	
Yes	59 (32)	34 (23)	25 (81)	
Clinic attendance				0.4
No	94 (27)	79 (26)	15 (33)	
Yes	250 (73)	220 (74)	30 (67)	

* Median (IQR); n (%).

† Wilcoxon rank-sum test; Pearson’s χ² test; Fisher’s exact test.

MELD, Model for End Stage Liver Disease.

In the unmatched cohort, overall mortality at 1 year was 19% and abstinence rate at 1 year was 47%.

### Matching

**Figure 1:**
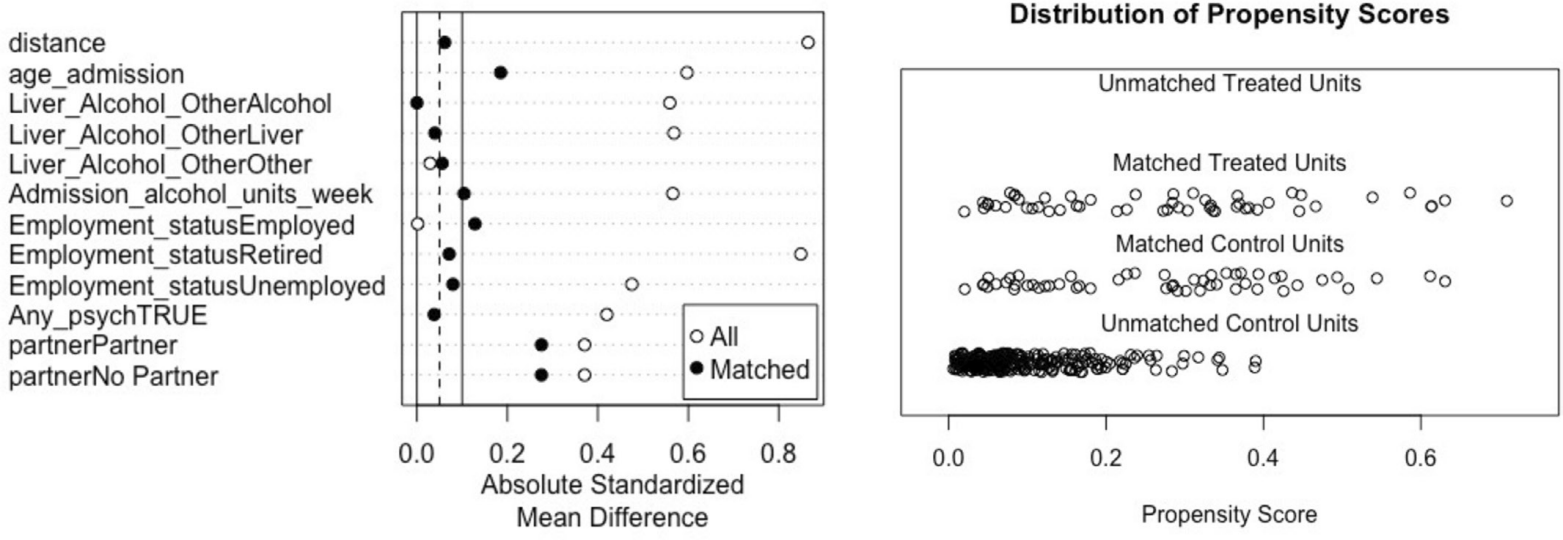
Balance of matched covariates before and after matching (Love plot, left) and distribution of propensity scores (right) in matched and unmatched cohorts.

Variables selected for the initial matching process were: age, marital status (partner or not), employment status (employed, retired, unemployed), alcohol intake on admission (units/week), psychiatric comorbidity, and reason for admission (liver, alcohol or other). These variables were all patient factors which showed statistically significant differences between cases and controls and were considered to have the potential to contribute to differences in acamprosate prescribing and outcomes. The inclusion of all these variables in the model produced a reasonable match result for all variables with standardised mean differences being<0.1 for most variables with the remaining variables all<0.3 (figure 1). After matching, there were no statistically significant differences in demographics between the acamprosate and non-acamprosate groups (table 2). Two cases with data missing for the variables used in matching were dropped as were all non-matched controls.

**Table 2:** Matched data

	Overall, n=106*	Controls, n=53*	Acamprosate, n=53*	P value†
Age	51 (45–58)	51 (48–58)	51 (43–57)	0.3
Female	31 (29)	13 (25)	18 (34)	0.3
Reason for admission				>0.9
Alcohol	56 (53)	28 (53)	28 (53)	
Liver	37 (35)	19 (36)	18 (34)	
Other	13 (12)	6 (11)	7 (13)	
Alcohol-associated hepatitis	25 (24)	14 (26)	11 (21)	0.5
Alcohol consumption (units/week)	145 (100–280)	140 (80–240)	150 (100–280)	0.6
Living status				0.093
Alone	44 (43)	26 (51)	18 (35)	
Not alone	59 (57)	25 (49)	34 (65)	
Partner				0.14
Partner	31 (29)	12 (23)	19 (36)	
No partner	75 (71)	41 (77)	34 (64)	
Employment status				0.8
Employed	25 (24)	11 (21)	14 (26)	
Retired	9 (8.5)	5 (9.4)	4 (7.5)	
Unemployed	72 (68)	37 (70)	35 (66)	
Psychiatric comorbidity	45 (42)	23 (43)	22 (42)	0.8
Length of stay (days)	4 (2–9)	4 (2–9)	4 (2–8)	0.6
MELD	17.0 (13.0–21.0)	17.0 (13.0–22.0)	17.0 (13.0–20.0)	0.5
Review by liaison psychiatry	91 (87)	43 (81)	48 (92)	0.1
Outcomes at 1 year				
No of admissions	2 (0–4)	1 (0–2.8)	3 (1–4.5)	<0.001
Readmitted	66 (71)	26 (57)	40 (85)	0.002
MELD	13 (11–17)	13 (11–17)	13 (11–17)	>0.9
Abstinence				0.4
Abstinent	23 (30)	13 (35)	10 (26)	
Not abstinent	53 (70)	24 (65)	29 (74)	
Mortality				>0.9
Dead	12 (13)	6 (12)	6 (13)	
Alive	84 (88)	43 (88)	41 (87)	
Community alcohol service use				<0.001
No	23 (40)	17 (65)	6 (19)	
Yes	34 (60)	9 (35)	25 (81)	
Clinic attendance				0.058
No	13 (23)	9 (36)	4 (13)	
Yes	43 (77)	16 (64)	27 (87)	

* Median (IQR); n (%).

† Wilcoxon rank-sum test; Pearson’s χ² test; Fisher’s exact test.

MELD, Model for End Stage Liver Disease.

### Matched cohort

After matching, at 1 year there was a statistically significant higher rate of readmission (85% vs 57%, p<0.001) and of repeat admissions (median 3 vs 1 p<0.001) in the acamprosate group. There were no statistically significant differences in abstinence rates or mortality at 1 year. In the matched cohort, mortality at 1 year was 13% and abstinence rate at 1 year was 30%. Patients prescribed acamprosate were much more likely to have also engaged with community alcohol services after discharge (81% vs 35% p<0.001). There was no difference in clinic attendance at 1 year between groups.

We analysed all repeat admissions to determine the reasons for readmission and assess for any adverse events related to acamprosate prescription (table 3). The readmission rate (patients with at least one readmission) was higher in all categories for the acamprosate group. This difference was significantly different for readmissions due to Alcohol (p<0.001) and Other causes (p=0.005) but not for readmissions due to decompensated liver disease or alcohol-associated hepatitis (p=0.196). Total numbers of repeat admissions were also higher in all categories (Liver, Alcohol, Other) for the acamprosate group. The distribution of admission types was not significantly different between groups (p 0.4), case notes review did not identify any readmissions specifically related to acamprosate use.

**Table 3:** Reasons for repeat admissions

	Overall, n=93	Controls, n=46	Acamprosate, n=47	P value*
Patients with at least one repeat admission†				
Total	66 (71)	26 (57)	40 (85)	0.002
Alcohol	47 (51)	15 (33)	32 (68)	<0.001
Liver	17 (18)	6 (13)	11 (23)	0.196
Other	29 (31)	8 (17)	21 (45)	0.005
Total numbers of repeat admissions†				
Total	257	94	163	
Alcohol	171 (67)	66 (70)	105 (64)	0.4
Liver	31 (12)	8 (8.5)	23 (14)	
Other	55 (21)	20 (21)	35 (21)	

Data on repeat admission was not available for 13 patients (7 Controls and 6 cases).

* Pearson’s χ² test.

† n (%).

## Discussion

It was anticipated that the prescription of acamprosate would be associated with a reduction in hospital admissions. In fact, acamprosate patients were readmitted more frequently and had more overall repeat admissions over the 1-year follow-up. Although we did not identify enough patients prescribed acamprosate to meet the requirements of our predetermined power calculation, the current recruitment of 53 cases would give the study an 80% power to detect a 25% difference in readmission at 95% significance. We have also accounted for several potential confounders in our matching process which might affect not only acamprosate prescription but also readmission rates. These included baseline alcohol intake, comorbid psychiatric diagnosis, reason for index admission, marital status, and employment status. The results are consistent with a national Swedish cohort study which showed an association between acamprosate monotherapy and increased AUD-related hospital admissions.^17^ In contrast, Tyson *et al*, noted lower hospital admissions associated with acamprosate compared with baclofen.^15^ Of note, patients in that study were mostly started on pharmacotherapies in the outpatient setting, whereas all included patients here were started on therapy during a hospital admission. This likely explains why the overall number and rate of repeat admissions in both cases and controls was higher in this study.

There are a number of potential explanations for this. It is likely that acamprosate was prescribed to patients with more severe forms of AUD. In this study we were not able to distinguish levels of alcohol dependence which could be done using validated tools or through formal psychiatric assessment. Patients with more severe disease would be more likely to relapse and more likely to report to the hospital. In the unmatched cohort, we noted a correlation between acamprosate prescription and higher rates of psychiatric comorbidity and poor social support. Patients who were prescribed acamprosate also had higher alcohol use in units/week. Finally, it is recognised that when studying interventions for AUD, one must consider that the prescription of intervention might denote severe dependency and thus predict a worse outcome.^18 19^

Local practice is for patients to be reviewed as an inpatient by a specialist Liaison Substance Misuse psychiatry team which consists of psychiatrists, nurse practitioners and alcohol workers. Local policy is also that AUD medications only be prescribed in conjunction with patient engagement with community alcohol services. We found much higher rates of engagement with community alcohol services in those prescribed acamprosate despite both groups having equal access to inpatient liaison psychiatry review. This could suggest that in the group who were not prescribed acamprosate, relapse prevention strategies were either declined or deemed not to be required.

Examining the whole cohort we noted that patients who were prescribed acamprosate were younger, and were more often admitted for reasons related to alcohol use rather than for decompensated liver disease. This finding perhaps reflects ongoing caution among prescribers when using these medications in patients with advanced liver disease. Acamprosate was not associated with any difference in mortality or MELD scores in survivors at 1 year. Although there was an increase in admissions, none were clearly attributable to acamprosate. Vannier *et al* found higher rates of decompensation in patients prescribed acamprosate.^16^ In this study, repeat related to decompensated liver disease were also slightly higher in the acamprosate group but this difference was not statistically significant. It might be that the beneficial effects of acamprosate are not evident in this population since the severity of either their liver disease or their AUD may mask any positive impact of the medication on clinical outcomes.

### Strengths and weaknesses

This study adds to the very limited data available on the safety and efficacy of acamprosate use in patients with alcohol-related cirrhosis and alcohol-associated hepatitis. By collecting granular data, including data on marital status, living situation and employment, we were able to control for these variables, something not always possible in larger database-driven cohort studies. Rather than relying on coding, a hepatologist individually reviewed case notes to improve the accuracy and validity of the data.

We did not collect data on compliance with medication in this study. Rather the data can be considered an intention-to-treat analysis, examining the real-world impact of the prescription of acamprosate to prevent relapse following hospital admission. Observational data suggests that good adherence can be expected in more than half of patients but that poor adherence is associated with an increased risk of alcohol-related mortality.^20^ It might be that specific compliance therapy should be considered as an adjunct to the prescription.^21^ Although we used detailed electronic health records to collect data, there are inherent weaknesses with retrospective data collection, including missing data. Moreover, this study reflects the experience of a single centre and results may not be generalisable.

## Conclusion

Acamprosate appears to be prescribed more often in younger patients, admitted due to the direct effects of alcohol intake rather than decompensation of liver disease, and who may be at higher risk of relapse due to social factors. After controlling for potential confounders through case–control matching, acamprosate was associated with higher rates of hospital readmission without any benefit in mortality or abstinence rates. It might be that in this population, acamprosate alone is insufficient to impact clinical outcomes in 1 year.

## Supplementary material

10.1136/bmjgast-2024-001654 online supplemental material 1

## Data Availability

All data relevant to the study are included in the article or uploaded as supplementary information.
